# Robust Target Detection and Tracking Algorithm Based on Roadside Radar and Camera

**DOI:** 10.3390/s21041116

**Published:** 2021-02-05

**Authors:** Jie Bai, Sen Li, Han Zhang, Libo Huang, Ping Wang

**Affiliations:** 1 Institute of Intelligent Vehicles, School of Automotive Studies, Tongji University, Shanghai 201804, China; baijie@tongji.edu.cn (J.B.); lisen@tongji.edu.cn (S.L.); hanzhang@tongji.edu.cn (H.Z.); 2 College of Electronic Science and Technology, Tongji University, Shanghai 201804, China; pwang@tongji.edu.cn

**Keywords:** target detection and tracking, sensor fusion, roadside radar and camera, intelligent transportation system

## Abstract

Intelligent transportation systems (ITSs) play an increasingly important role in traffic management and traffic safety. Smart cameras are the most widely used sensors in ITSs. However, cameras suffer from a reduction in detection and positioning accuracy due to target occlusion and external environmental interference, which has become a bottleneck restricting ITS development. This work designs a stable perception system based on a millimeter-wave radar and camera to address these problems. Radar has better ranging accuracy and weather robustness, which is a better complement to camera perception. Based on an improved Gaussian mixture probability hypothesis density (GM-PHD) filter, we also propose an optimal attribute fusion algorithm for target detection and tracking. The algorithm selects the sensors’ optimal measurement attributes to improve the localization accuracy while introducing an adaptive attenuation function and loss tags to ensure the continuity of the target trajectory. The verification experiments of the algorithm and the perception system demonstrate that our scheme can steadily output the classification and high-precision localization information of the target. The proposed framework could guide the design of safer and more efficient ITSs with low costs.

## 1. Introduction

Road traffic safety and efficiency are the key challenges in modern transportation. According to the Global Status Report on Roads in 2018, the number of road traffic deaths is over 1.35 million per year. More than half of all deaths are among vulnerable road participants: cyclists, motorcyclists, and pedestrians [1]. Intersection collisions account for over 40% of total traffic accidents, which not only seriously threaten people’s lives, but also cause severe traffic congestion [2]. Research has shown that over 60% of these collisions can be avoided if drivers receive a warning just half a second in advance [3,4]. To improve traffic problems and build smart cities, the intelligent transportation system (ITS) has been widely studied [5,6]. Especially in recent years, with the rapid development of 5G communication technology, artificial intelligence, sensor technology, and high-performance chip technology, the related technology of ITS has exploded, such as intelligent connected vehicles (ICVs) [7], vehicle-to-everything (V2X) [8,9], and edge sensing and computing [10,11]. Over-the-horizon perception for ICVs based on V2X and intelligent roadside units (RSUs) can be used for collision prevention at intersections to improve traffic safety [12,13]. Moreover, the essential task of intelligent RSUs is to build a stable and reliable perception system.

The roadside perception unit (RPU) uses cameras, lidars, and millimeter-wave (MMW) radars to detect and locate targets within the field of view. Due to the advancement of sensor technology and perception algorithms, research on roadside perception solutions can be divided into two phases. In early research, before 2011, the focus was on low-beam lidar and traditional vision-processing methods. H. Zhao et al. used multiple laser scanners located at different locations to form an observation network for intersection monitoring [14]. Wang C. at el. proposed a move-stop hypothesis tracking approach to solve the move-stop-move maneuvers by using the single-line lidar [15]. Daniel Meissner et al. utilized multiple four-layer laser scanners mounted at high parts of the infrastructure to detect and track objects inside intersections [16]. Oliver, N. M. et al. proposed a multilevel tracking approach that combined low-level image-based blob detection and high-level Kalman filtering for multi-target tracking at intersections [17]. Peyman B. developed a vision-based surveillance system for vehicle counting and tracking in an intersection by using a background-modeling technique [18]. Due to the lack of performance in target classification, radar was mainly used for monitoring the speed and distance of road vehicles during this time [19,20]. For a more stable and robust perception system, researchers have proposed some schemes to improve the target detection and tracking performance by using laser and camera fusion [21] or radar and camera fusion [22].

In the last decade, the performance of perception systems has been enormously improved with rapid developments of high-beam lidars, high-resolution radars, and deep-learning technologies. The current state-of-the-art algorithms based on convolutional neural networks (CNNs), such as yolo-V4 [23] and EfficientNet [24], can offer both high processing speed and detection accuracy. The problem of detecting small targets at a far distance also has been greatly improved. Shuai Hua et al. built a multi-vehicle tracking framework based on the Yolo network that can be used for real-time traffic applications [25]. Some scholars have also tried to use advanced image-processing methods to estimate the vehicle–pedestrian collision probability [26] or detect abnormal events [27] at intersections. The new generation of lidar has a 360-degree scanning field of view and more scanning beams, such as 32 lines, 64 lines, and 128 lines, which can provide higher detection accuracy. J. Z. et al. achieved tracking and speed estimation of vehicles at intersections using 32-line lidar with a speed estimation accuracy of 0.22 m/s [28]. Z. Z. et al. achieved large-area scenario modeling and high-resolution target tracking at intersections using 3D point clouds [29]. Some authors have also implemented real-time queue range detection [30] and collision risk analysis [31] based on roadside lidar. Similarly, the new generation of 79 GHz ultra-bandwidth radar overcomes the lack of angular resolution and is also widely used in ITS. W. L. et al. proposed a classification algorithm for pedestrians and vehicles at intersections based on point clouds of 79 GHz radar [32]. Some scholars have built safety systems for vulnerable road users [33] and traffic intersection surveillance systems [34,35] at intersections based on 79 GHz radar and V2X technology. In order to improve the robustness of RPU, some scholars have proposed methods based on radar and camera fusion for vehicle detection and width estimation in bad weather [36,37]. Christoph S. et al. proposed a two-stream CNN method for auto-calibration of a radar and camera to realize robust detection of vehicles on the highway [38]. Kaul P. at al. presented a weakly supervised multiclass semantic segmentation network to achieve semantic segmentation of multichannel radar scan inputs with the help of a camera and lidar segmentation system [39].

In the studies of and developments in ITSs, the main purpose is to improve traffic safety and efficiency. Therefore, an important prerequisite for all related research, such as traffic monitoring, behavior prediction, and collision warning, is to establish a robust target detection and tracking system. In addition to the detection accuracy, we also need to consider the cost of the whole solution for large-scale deployment [4]. In [40], the authors evaluated sensors using five criteria: range, resolution, contrast, weather, and cost. In ideal conditions, the vehicle-detection range can reach up to 100 m, and the pedestrian-detection range can reach up to 43 m using a 16-line lidar [29]. Although a higher beam lidar can increase detection accuracy and effective range, the high cost of lidar hinders its large-scale market application. According to the investigation in [40], the cost of a 16-line lidar is more than 10 times that of a 77 GHz millimeter-wave radar or a mono-camera, and the price of a 64-line lidar is between USD 40,000 and 70,000. Cameras are currently the most widely used sensor, and image-processing algorithms based on deep learning have evolved tremendously. However, the performance of the lidar and camera suffers a large degradation in bad weather scenarios [41,42] Radar has weather robustness and doppler velocity sensitivity, but its angular resolution is insufficient [43]. Cameras and radars can complement strengths and weaknesses in several aspects. Their low cost is also a hot spot of current research.

In this study, we developed a stable RPU for the detection and real-time localization of traffic participants. We hope to adopt a low-cost solution based on radar and camera fusion to realize high localization accuracy close to lidar, which can contribute to large-scale applications. There are two major problems in radar and camera perception [43]. The first is measurement loss and noise interference in complex traffic scenarios. The limited performance of the detection algorithm, target occlusion, and environmental noise can cause missed detections and false alarms. The second is the limited localization accuracy. The longitudinal range accuracy of the camera and the lateral range accuracy of the radar decrease significantly during the localization of targets at far distances. Therefore, the contribution of this paper is to propose an optimal attribute fusion algorithm, which is a detection-tracking algorithm based on the Gaussian mixture probability hypothesis density (GM-PHD) framework [44]. We introduce lost labels and attenuation functions to adaptively maintain the target life cycle and achieve continuity of the tracking trajectory.

The structure of this paper is organized as follows. Section 2 introduces the related work of target detection and data processing. Then, in Section 3, we describe the proposed optimal attribute fusion tracking algorithm. Section 4 analyzes and discusses the experimental results. Finally, Section 5 summarizes the conclusions of this paper and future works.

## 2. Preliminaries

In this section, we mainly introduce the detection principle and calibration process of radar and camera, as well as the pre-processing method for data fusion.

### 2.1. Target State Vector and Motion Model

Consider a traffic scenario at an intersection, with vehicles, pedestrians, cyclists, and other targets moving on the roadway. Let *M*(*k*) denote the number of targets at time *k*, and the motion state of targets can be represented as the state set $X_{k}{\;=\;\{x}_{1,k},\;\cdots {,\;x}_{i,k},\;\cdots {,x}_{M(k),k}\}$. The state vector $x_{i,k}$ describes the position and velocity of target *i* at time *k* and is defined as:

(1) $$x_{i,k}={\left[x\;\;y\;\;v_{x}\;\;v_{y}\right]}^{T},\;\;i\in M(k)$$

Let *F_k_* be the state transfer matrix, and the movement of the target follows the motion of Equation (2):

(2) $$x_{k+1|k}=F_{k}x_{k}+\xi_{k}$$

In the RPU, the sensor is often mounted on a light pole and has a fixed view field. All traffic participants appear and disappear independently of the sensor’s field of view. Targets can be captured if they are within the sensor’s range of perception. If the number of targets observed by the sensor is *N*(*k*) at time *k*, then all observed targets can be represented by the measurement set $Z_{k}{\;=\;\{z}_{1,k},\;\cdots {,\;z}_{j,k},\;\cdots {,z}_{N(k),k}\}$. The observation vector $z_{i,k}$ is an imperfect measurement of the state of the observed target *j* at time *k*, having the same form as $x_{i,k}$. The sensor’s observation model is described as:

(3) $$z_{k}=H_{k}x_{k}+\varsigma_{k}$$

where *H_k_* is the observation matrix of the linear dynamic system, and $\xi_{k}$ and $\varsigma_{k}$ are system and observation white Gaussian noise with covariance $N\left(\xi ;0,R\right)$ and $N\left(\varsigma ;0,R\right),$ respectively. Note that the state set and observation set of the target have no correspondence and order, and *M*(*k*) is not equal to *N*(*k*) due to clutter interference and occlusion. Our task is to estimate the number of targets and their state from the multiple observation set.

The state and observation set of targets are considered to be random finite sets (RFS), and the number of targets in the set varies with time and has no regular ordering [45].

(4) $$\begin{array}{l} X_{k}=\left\{x_{1,k},x_{2,k},\cdots ,x_{M(k),k}\right\}\in F(X)\\ Z_{k}\;\;=\;\;\left\{z_{1,k},z_{2,k},\;\cdots ,\;\;z_{N(k),k}\;\right\}\in F(Z) \end{array}$$

where $X_{k}$ and $Z_{k}$ are subsets of $F\left(X\right)$ and $F\left(Z\right)$, respectively, and $F\left(X\right)$ and $\;F\left(Z\right)$ are the set of all finite subsets of state space X and measurement space Z, respectively. The task of MTT is to estimate the state of targets from the sensor observations. Based on the theory of RFS, the multi-target tracking can be regarded as a filtering problem with state space $F\left(X\right)$ and observation space $\;F\left(Z\right)$. Generally, the number of elements in the state set is smaller than the observation set, i.e., $N_{\left(k\right)}\leq M_{\left(k\right)}$.

### 2.2. Radar Detection Model

**Figure 1:**
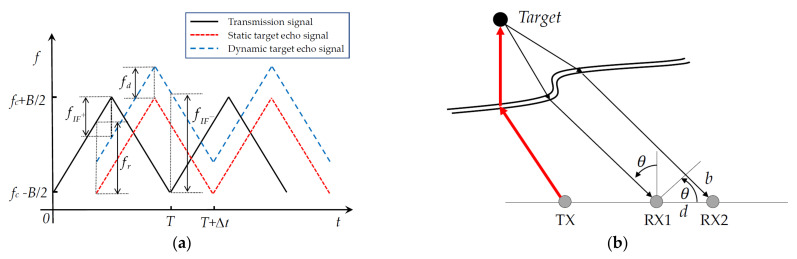
(**a**) Linear frequency modulation continuous wave (LFMCW) radar signal waveform processing; (**b**) principle of azimuth measurement of the target. TX is the transmitting antenna, and RX is the receiving antenna.

MMW radar directionally transmits electromagnetic radio frequency signals and analyzes the echo signals of surroundings to detect targets. By measuring the time delay and phase shift of the echo signal, the distance and velocity of the target can be measured. Directional antennas or phase comparison techniques can determine the azimuth of the target [46]. As shown in Figure 1a, the echo signal produces a time delay due to the propagation of electromagnetic waves between the radar and the target, resulting in a distance frequency shift. For dynamic targets, in addition to the distance frequency shift *f_d_*, the target movement also produces Doppler frequency shift *f_r_*. The transmission signal and the echo signal produce two differential frequencies *f_IF_^+^* and *f_IF_^-^* on the rising and falling edges of the frequency, and *f_IF_*^+^ = *f_r−_f_d_*, *f_IF_*^+^ = *f_r_* + *f_d_*. The range *R* and velocity *v* of a target can be calculated by the following equation:

(5) $$R=\frac{T\times c}{8B}(f_{IF^{+}}+f_{IF^{-}})$$

(6) $$v=\frac{c}{4f_{c}}(f_{IF^{+}}-f_{IF^{-}})$$

where *T* and *B* are the period of frequency modulation and modulation bandwidth, respectively; *f* is the center frequency of the transmission waveform; and *c* is the speed of light.

The azimuth is estimated using the phase-comparison method, as shown in Figure 1b. The target signal has a travel distance during propagation, and thus a corresponding phase difference in the echo signal. The azimuth *θ* of the target is calculated as shown in Equation (7):

(7) $$\theta =\arcsin(\frac{\lambda w}{2\pi d})$$

where *w* is the phase difference caused by the distance difference of the target echo signal, *d* is the distance between antennas RX1 and RX2, and λ is the wavelength. Therefore, the state of the target *i* can be represented as a vector $z_{i}^{R}=\left[R^{R},v^{R},\theta^{R}\right]$. After a series of data processing, radar can output sparse point-cloud information for the dynamic and static targets of the surroundings. The radar measurement set is $Z^{R}{=\{z}_{1}^{R},\cdots {,z}_{i}^{R},\cdots {,z}_{M}^{R}\}$, and the observation equation at time *k* is as follows:

(8) $$Z_{k}^{R}=H^{R}X_{k}+W_{k}^{R}(R,\theta )$$

where *H^R^* is the radar observation vector and $W_{k}^{R}$ is observation noise, which is related to radar characteristics and environmental factors. MMW radar has high range accuracy and Doppler velocity accuracy. However, radar lacks capabilities in angle measurement and target classification, which can be compensated with the help of cameras.

### 2.3. Camera Detection Model

**Figure 2:**
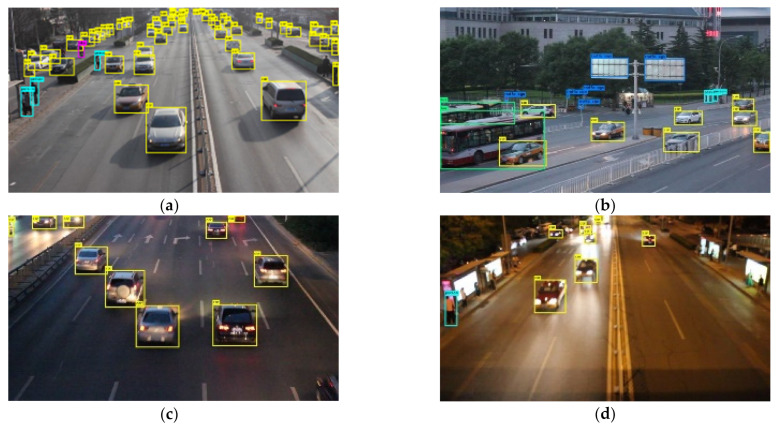
Partial test results for different scenarios of the UA-DETRAC dataset. (**a**) Sunny daytime; (**b**) cloudy evening; (**c**) night; (**d**) interference by strong light at night.

Cameras are the most widely used in intelligent vehicles and ITSs, providing rich scenario information such as target classification [47], lane lines [48], traffic signs [49], etc. Compared to automotive cameras, roadside cameras are mounted higher and have a wider observation field. The main challenges for roadside cameras are the dynamics of the background and the detection of small targets. In recent years, the rapid development of deep-learning-based image-processing algorithms has dramatically improved accuracy and real-time target-detection performance. In this paper, we retrained the YoloV4 framework, which is a one-stage target-detection algorithm, to perform image-based target detection and localization tasks [23]. Yolov4 offers a good balance of detection precision and detection speed, making it a suitable edge-computing platform for roadside units. To ensure the detection precision, we performed migration training on the roadside traffic dataset UA-DETRAC to obtain the training weights for the roadside scenario [50]. UA-DETRAC is a dataset for multi-objective detection based on real urban traffic conditions collected in China. The dataset contains a variety of traffic scenarios (urban highways, intersections, flyovers, and elevated gate crossings) under different weather conditions (sunny, rainy, and cloudy), as well as for different time periods (daytime and nighttime), covering typical Chinese urban roads. The partial test results after retraining are shown in Figure 2. The test results show that the algorithm can implement pedestrian and vehicle detection, and detect small targets over long distances in clear daylight. However, as shown in Figure 2d, some vehicles and pedestrians were not detected due to interference from the intense background light. The image detection results are the bounding box and classification information of targets. The spatial position information of the target can be obtained by converting the pixel coordinate system to the geodetic coordinate system through camera calibration, which will be described in the next section. The velocity of the target can be estimated by the position difference between adjacent frames. Similarly, the state of the target *i* can be also represented as a vector $z_{i}^{C}=\left[R^{C},v^{C},\theta^{C}\right]$. The camera measurement set is $Z^{C}{=\{z}_{1}^{C},\cdots {,z}_{i}^{C},\cdots {,z}_{N}^{C}\}$, and the observation equation at time *k* is expressed in a form similar to Equation (9) as:

(9) $$Z_{k}^{C}=H^{C}X_{k}+W_{k}^{C}(R,\theta )$$

where $H^{C}$ is the camera observation vector and $W_{k}^{C}\;$ is observation noise. Compared to MMW radar, the advantage of the camera is the classification of the target and the estimation of the azimuth, but the disadvantage is the lack of accuracy in the estimation of range and velocity.

### 2.4. Sensor Calibration

**Figure 3:**
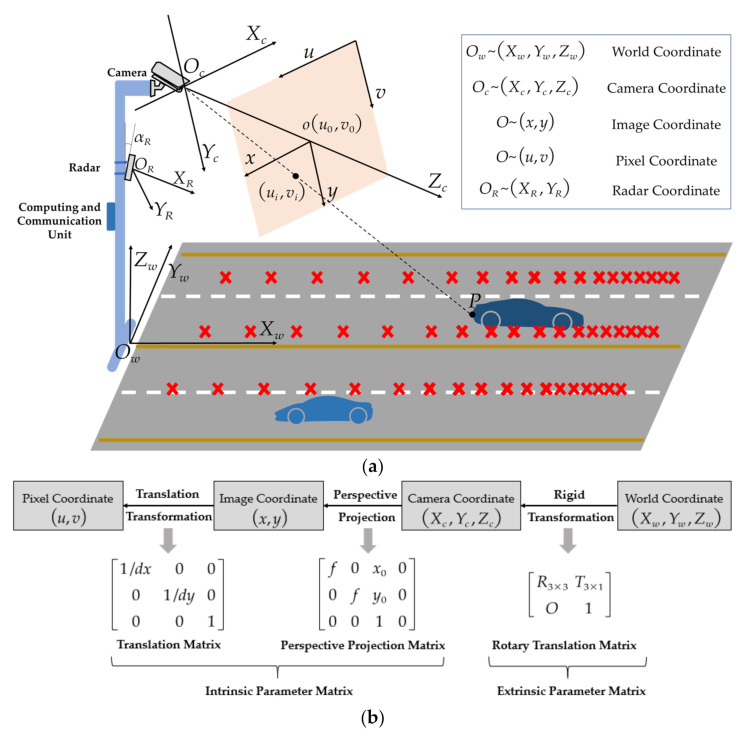
(**a**) Joint calibration method of roadside cameras and millimeter-wave (MMW) radar; (**b**) coordinate mapping from the world coordinate system to the pixel coordinate systems.

In this work, we performed target detection and tracking based on the fusion of roadside radar and camera. With the joint calibration of the roadside camera and radar, as shown in Figure 3a, the radar and camera detection results can be fused with data in the same world coordinate system. According to the calibration process, the calibration parameters of the camera can be divided into an intrinsic parameter matrix and an extrinsic parameter matrix, and the mapping relationship is as follows:

(10) $$Z_{c}\left[\begin{array}{l} u\\ v\\ 1 \end{array}\right]=\left[\begin{array}{cccc} f_{x} & 0 & u_{0} & 0\\ 0 & f_{x} & v_{0} & 0\\ 0 & 0 & 1 & 0 \end{array}\right]\;\cdot \;\left[\begin{array}{cc} R & T\\ 0 & 1 \end{array}\right]\left[\begin{array}{l} x_{w}\\ y_{w}\\ z_{w}\\ \;\;1 \end{array}\right]$$

where the first to the right of the Equation (10) is the camera’s intrinsic parameter matrix; *f_x_* and *f_y_* are the scale factors of the camera on the *u* and *v* axes, *f_x_ = f*/*dx*, *f_y_* = *f*/*dy*. $(u_{0},v_{0}$) is the optical center of the camera, $u_{0}{\;=\;x}_{0}{/d}_{x}{,\;v}_{0\;}{=\;y}_{0}{/d}_{y}$. Most cameras have radial and tangential distortion, which are also intrinsic parameters of the camera that need to be calibrated. The intrinsic parameters of the camera were calibrated using the classical calibration method [51]. *R* and *T* are the rotation matrix and translation matrix, respectively, which are extrinsic parameters of the camera related to the camera’s mounting position and angle [52]. If the camera’s yaw angle and roll angle relative to the world coordinate system are set to zero, the extrinsic parameter matrix can be represented by the camera’s optical center height *H* and pitch angle $\alpha_{c}$. The vanishing point calibration method can be used to calibrate the extrinsic parameters of the camera [53].

The scanning range of the radar is a conical region. To guarantee a high accuracy of the radar at its observation range, only the installation height h and pitch angle $\alpha_{R}$ need to be considered. and the conversion from the radar to the world coordinate system is:

(11) $$\left[\begin{array}{l} R_{w}\\ v_{w}\\ \theta_{w} \end{array}\right]=\left[\begin{array}{c} R^{R}\cos\alpha_{R}\\ v^{R}\cos\alpha_{R}\\ \theta^{R} \end{array}\right]$$

Since the radar and image are installed at different positions, and the measurement error increases with distance, a joint calibration is necessary for the common observation area. The sensor measurements require an additional correction to calibrate the measurement error, which can be accomplished using ground calibration points for simultaneous calibration, as shown by the red cross in Figure 3a. Corrections between calibration points can be estimated by interpolation, and the distance between the calibration points should decrease as the measurement distance increases. Although the calibration increases the workload, it is indeed important and can significantly improve the accuracy of target estimates. Ultimately, radar and image observations can be unified into a single world coordinate system for data fusion.

### 2.5. Data Pre-Correlation

According to the sensor detection model constructed in Section 2.2 and Section 2.3, the MMW radar has higher accuracy in range and Doppler velocity. At the same time, the camera has more advantages in target classification and azimuth. Before tracking, the measurement sets of two sensors are pre-correlated by using the nearest-neighbor correlation method. Using radar-range measurements and image-angle measurements to update the target state can improve localization accuracy. The pre-correlation results of sensor measurements at time *k* can be divided into fusion measurement $Z_{k}^{f}$, uncorrelated camera measurement $Z_{k}^{C},$ and uncorrelated radar measurement $Z_{k}^{R}$, forming a new measurement set $Z_{k}^{\mathrm{New}}{=\;\{Z}_{k}^{f}{,Z}_{k}^{C}{,\;Z}_{k}^{R}\}$. The new state vector of the target can be described in terms of position, velocity, and classification $x\;=\;[x\;\dot{x}\;y\;\dot{y}\;\xi ]$:

(12) $$x_{k}^{i}=\left[\begin{array}{c} x\\ \dot{x}\\ y\\ \dot{y}\\ \xi \end{array}\right]=\left[\begin{array}{c} R_{w}\cos\theta^{C}\\ v_{w}\cos\theta^{C}\\ R_{w}\sin\theta^{C}\\ v_{w}\sin\theta^{C}\\ \xi \end{array}\right]$$

where ξ represents the classification of target detected by camera. For ease of computer processing, the target’s classification can be denoted by a number, e.g., “pedestrian: 1, vehicle: 2, cyclist: 3, unassociated target: 0”.

## 3. System Overview

For real-time monitoring and security purposes, the ITS system needs to obtain continuous and accurate state information of surrounding targets in the observation area. To improve the accuracy and stability of target detection and tracking, we proposed an optimal attribute fusion algorithm based on the GM-PHD algorithm framework [44]. The GM-PHD tracking framework estimates the state and number of targets simultaneously, which is suitable for the complex traffic scenarios in which the number of targets changes over time. In our improvement strategy, the idea is to build loss tags and attenuation function to achieve the continuity of the target trajectory and use the optimal measurement to improve localization accuracy. The improved tracking algorithm is introduced in the following five processes: initialization, prediction, update, pruning, and merging.

### 3.1. Initialization

In the GM-PHD algorithm, Gaussian components are used to represent targets or potential targets. A Gaussian component $\;\left\{w,m,P,\varepsilon \right\}$ is expressed by weight *w*, mean state *m*, covariance *P*, and classification  ε. The state distribution of Gaussian components is described by a Gaussian mixture probability density function in the observation space, and the initial probability intensity $v_{0}$ is:

(13) $$v_{0}(x)=\overset{J_{0}}{\underset{i=1}{\sum }}w_{0}^{i}N\left(x;m_{0}^{i},P_{0}^{i}\right)$$

where $J_{0}$ is the number of targets or Gaussian components at initial moment; N(·;·,·) denotes the distribution of Gaussian component *i* with weight $w_{0}^{i}$, mean state $m_{0}^{i}$, and covariance matrix $P_{0}^{i}$. The classification ε of the target is not changed once it has been defined. New birth targets may appear with each measurement, and the target intensity function of new targets at time *K* is given by:

(14) $$\gamma_{k}(x)=\overset{J_{\gamma ,k}}{\underset{i=1}{\sum }}w_{\gamma ,k}^{i}N\left(x;m_{\gamma ,k}^{i},P_{\gamma ,k}^{i}\right)$$

where the covariance matrix $P_{\gamma ,k}^{i}$ describes the spread of the birth intensity near the peak $m_{\gamma ,k}^{i}$. The weight $w_{\gamma ,k}^{i}$ indicates the expected number of new targets from $\;m_{\gamma ,k}^{i}$, and the weight initialization function is given by:

(15) $$w_{\gamma ,k}^{i}=0.25N\left(x;m_{\gamma }^{1},P_{\gamma }^{1}\right)+0.25N\left(x;m_{\gamma }^{2},P_{\gamma }^{2}\right)+I_{k}^{i}$$

where $m_{\gamma }^{1}$ and $m_{\gamma }^{2}$ can be set as the focused point in the observation scene. If the new birth target is closer to the focal point, the higher the weighting coefficient is. $I_{k}^{i}$ is the bias coefficient, which is determined according to the correlation results of radar and image measurement sets. If the measurement $x_{\gamma ,k}^{i}$ is a fusion measurement, $I_{k}^{i}=w_{fu}$, otherwise $I_{k}^{i}=0$. The measurement loss of the target is unpredictable, and each Gaussian component is assigned a loss tag to count the number of times the measurement is lost. For identified targets, measurement loss may occur due to occlusion or undetected by the sensor. So, a loss tag $L_{loss}$ is used to count the measurement loss times of the target, which will be introduced in the update step. Finally, the initialization result of a target is $\left\{w,m,P,\varepsilon ,L_{loss}\right\}$, and $L_{loss}=0$ at initial moment.

### 3.2. Prediction

The posterior intensity at time *k* − 1 is a Gaussian mixture form, and the predicted intensity for time *k* is also a Gaussian mixture form, which consists of the survival target intensity $v_{S,k|k-1}$ and the new birth target intensity $\;\gamma_{k}\left(x\right)$. In this step, ε and $L_{loss}$ remain unchanged.

(16) $$v_{k-1}(x)=\overset{J_{k-1}}{\underset{i=1}{\sum }}w_{k-1}^{i}N\left(x;m_{k-1}^{i},P_{k-1}^{i}\right)$$

(17) $$v_{k|k-1}(x)=v_{s,k|k-1}(x)+\gamma_{k}(x)$$

where the $\gamma_{k}\left(x\right)$ is given in Equation (9), and the $v_{S,k|k-1}$ is given by:

(18) $$v_{S,k|k-1}(x)=\overset{I_{S,k|k-1}}{\underset{i=1}{\sum }}w_{S,k|k-1}^{i}P_{S,k}N\left(x;m_{S,k|k-1}^{i},P_{S,k|k-1}^{i}\right)$$

where $P_{S,k}$ is target survival probability, which is difficult to predict directly in the actual tracking scenario. Under the condition of low-speed target or high sensor sampling, the state transfer can be approximately considered as a linear Gaussian process.

(19) $$w_{k|k-1}^{i}=w_{k-1}^{i}$$

(20) $$m_{k|k-1}^{i}=F_{k-1}m_{k-1}^{i}$$

(21) $$P_{k|k-1}^{i}=Q_{k-1}+F_{k-1}P_{k-1}^{i}F_{k-1}^{T}$$

### 3.3. Update

The posterior density update also satisfies the Gaussian mixture distribution, consisting of a detected part and an undetected part:

(22) $$v_{k}(x)=\left(1-P_{D,k}\right)v_{k|k-1}(x)+\underset{z\in Z_{k}}{\sum }v_{D,k}\left(x;z\right)$$

where $P_{D,k}$ is the detection probability, which cannot be accurately estimated like $P_{S,k}$. $v_{D,k}$ is the posterior density of the detected part. $\left(1-P_{D,k}\right)v_{k|k-1}\left(x\right)$ indicates that the undetected target is updated with a state prediction instead of the posterior update. A method for joint estimation of clutter distribution and detection probability was proposed in [54]. However, this approach cannot directly solve the problem of measurements loss of targets. To simplify the update process without directly estimating $\;P_{S,k}$ and $P_{D,k}$, the elliptical gating method was introduced in this study, inspired by ideas in [55,56]. Assume that the residual vector of Gaussian terms corresponding to the *i*-th observation value and the *j*-th prediction value is $\;\varepsilon^{ij}$, and the corresponding covariance matrix is $S^{j}$.

(23) $$\varepsilon^{ij}=z_{k}^{i}-H_{k}\left(x_{k|k-1}^{j}\right)$$

(24) $$S_{k}^{j}=H_{k}P_{k}^{j}H_{k}^{T}+R_{k}$$

Then the discriminant of the elliptic threshold can be expressed as:

(25) $${\left(\varepsilon^{ij}\right)}^{T}{\left(S^{j}\right)}^{-1}\left(\varepsilon^{ij}\right)⩽T_{g}$$

where *T_g_* is the threshold. Some studies have also proposed adaptive threshold methods to improve performance [55]. According to Equation (25), the predicted Gaussian component can be divided into two parts: measurement existence and measurement loss, denoted by ${\left\{w,m,\;P,\varepsilon ,L_{loss}\right\}}_{j=1}^{J_{match}}\in Z_{match}$ and ${\left\{w,m,\;P,\varepsilon ,L_{loss}\right\}}_{j=1}^{J_{loss}}\in Z_{loss}$, respectively. So, the update process consists of two parts: the detection update and the missed detection update.

For the detection update, the target is detected by the sensor at the next moment, i.e., $P_{D,k}=1$. The posterior intensity of Gaussian components is given by:

(26) $$v_{k|k}(k)=\underset{z_{i}\in Z_{\mathrm{match}}^{\varepsilon }}{\sum }\overset{J_{\mathrm{match}}}{\underset{j=1}{\sum }}w_{k}^{j}\left(z_{i}\right)N\left(x;m_{k|k}^{j}\left(z_{i}\right);P_{k|k}^{j}\right)$$

where the update equation for weight *w*, mean state *m*, and covariance *P* is as follows:

(27) $$w_{k}^{j}\left(z_{i}\right)=\frac{w_{k|k-1}^{j}q_{k}^{j}(z_{i})}{\kappa_{k}(z_{i})+\overset{I_{\mathrm{match}}}{\underset{j=1}{\sum }}w_{k|k-1}^{j}q_{k}^{j}(z_{i})}$$

(28) $$m_{k|k}^{j}\left(z_{i}\right)=m_{k|k-1}^{j}+K_{k}^{j}\left(z-H_{k}m_{k|k-1}^{j}\right)$$

(29) $$q_{k}^{j}\left(z_{i}\right)=N\left(z_{i};H_{k}m_{k|k-1}^{j},R_{k}+H_{k}P_{k|k}^{j}H_{k}^{T}\right)$$

(30) $$P_{k|k}^{j}=\left[I-K_{k}^{j}H_{k}\right]P_{k|k-1}^{j}$$

(31) $$K_{k}^{j}=P_{k|k-1}^{j}H_{k}^{T}{\left[S_{k}^{j}\right]}^{-1}$$

Equations (24)–(31) are the recursive equations of the detection part. Note that in the weight update equation (27), the detection probability $P_{D,k}$ is removed because $P_{D,k}=1$ in this case. The classification of Gaussian components remains unchanged, and the loss tag $L_{loss}=0$.

**Figure 4:**
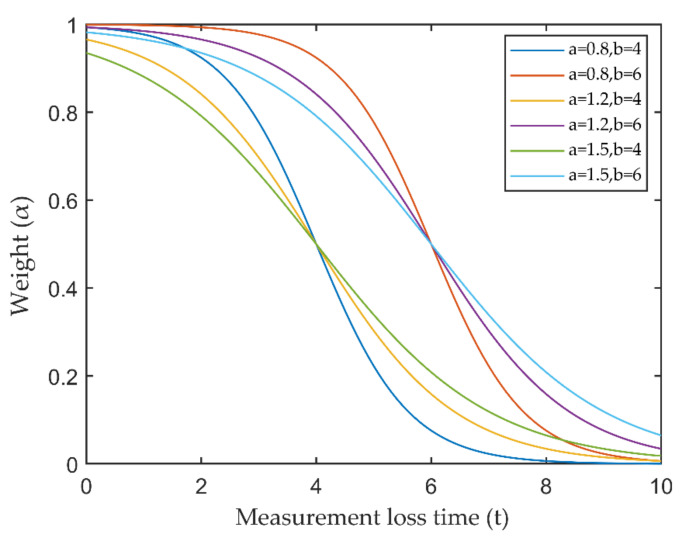
Visualization of weight attenuation function.

For the missed detection update, the target is not detected by the sensor at the next moment, i.e., $P_{D,k}=0$, and the idea of using predicted values for status updates continues to be followed. If target *j* loses measurement of the next time at time *k*, the corresponding loss tag value is increased by 1, i.e., $L_{loss,k|k}^{j}=L_{loss,k|k-1}^{j}+1$. However, not all Gaussian components need to be preserved, and only the targets of interest are worth maintaining. The parameter ε can be used to help select valid targets. Gaussian components with ε=1, 2, or 3, indicating that the targets are of concern to us, must be maintained. Gaussian components with  ε=0 represent other noise that can be dropped. Since the weight is essential for determining the survival and extinction of Gaussian components, targets with measurement loss should be given an attenuation function:

(32) $$w_{k|k}^{j}=\alpha_{k}^{j}(t)w_{k|k-1}^{j}$$

(33) $$m_{k|k}^{j}=m_{k|k-1}^{j}$$

(34) $$P_{k|k}^{j}=P_{k|k-1}^{j}$$

where $\alpha_{k}^{j}\left(t\right)$ is the attenuation function, and $t=L_{loss,k}^{j}$. In practical applications, the attenuation function can be selected according to our needs, and the Fermi–Dirac function was chosen in this study:

(35) $$\alpha_{k}^{j}(t)=\frac{1}{1+\exp((t-b)/a)}$$

where *a* and *b* are the parameters that determine the shape of the attenuation function, as shown in Figure 4. However, the targets of missed detection cannot be maintained forever, and the maximum cycle can be limited by the parameters *a* and *b* and the threshold together. For the re-identification problem of lost targets, the elliptic threshold of Equation (25) can be increased to ensure the stability of tracking.

### 3.4. Pruning and Merging

The pruning and merging process is a key step in extracting the target and reducing the ineffective Gaussian component. The computational complexity of the heuristic pruning and merging algorithm is $\;O\left(n_{k}{\left|Z_{k}\right|}^{3}\right)$ at each step [54]. In complex scenarios with much background noise, interference measurements consume a large amount of computational resources. Let us consider a simplified pruning process. The updated Gaussian components can be thought of as a state matrix as follows:

(36)    Upgrade  Zf  ZC ZR  Jmatch  Jno_matchXk|k−11Xk|k−12⋮Xk|k−1Jmatch⋮Xk|k−1Jno_match→[Xk(1,1)Xk(2,1)⋮Xk(Jmatch,1)⋮Xk|k−1(Jno_match,1)⋯⋯⋱⋯⋱⋯Xk(1,Zf)Xk(2,Zf)⋮Xk(Jmatch,Zf)⋮Xk|k−1(Jno_match,Zf)⋯⋯⋱⋯⋱⋯Xk(1,ZC)Xk(2,ZC)⋮Xk(Jmatch,ZC)⋮Xk|k−1(Jno_match,ZC)⋯⋯⋱⋯⋱⋯Xk(1,ZR)Xk(2,ZR)⋮Xk(Jmatch,ZR)⋮Xk|k−1(Jno_match,ZR)],

Then, the weights of each Gaussian component can be normalized by column to produce a weight matrix, which is the same as the state matrix. The state matrix and weight matrix are sparse matrices due to applicating the elliptical gating method. Assuming that each measurement has a single source, i.e., that measurement is generated by only one target. Then, each column needs to extract only one Gaussian component with the highest weight in the detection part. For the missed detection part, only Gaussian components of ε=1,2, or 3 need to be retained. Finally, Gaussian components greater than the threshold are selected as the final estimation targets.

## 4. Experiment and Results

In this section, we discuss and analyze the experimental results conducted in typical traffic-intersection scenarios to evaluate the localization and perception performance of the proposed algorithm. We also demonstrated the application of the proposed algorithm in the RSU and On board Unit (OBU) platform at the system level.

### 4.1. Experiment Platform and Configuration

**Figure 5:**
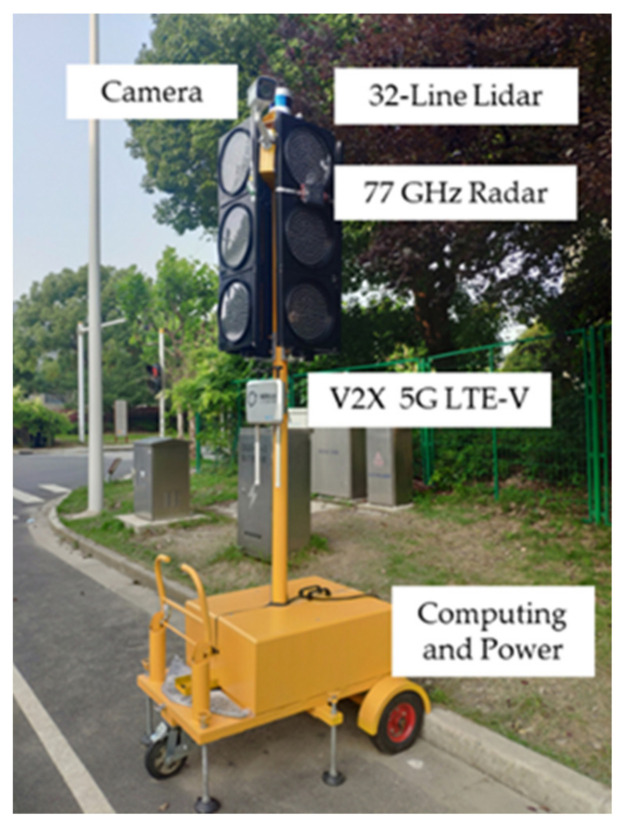
The movable intelligent roadside sensing and computing platform. The sensor unit consists of a radar, a camera, and a 32-line lidar; the vehicle-to-everything (V2X) unit uses the 5G LTE-V communication format; and the computing unit is an Nvidia Xavier.

In our study, we built a movable intelligent roadside sensing and computing platform that can be used for multiple scenario testing. The roadside platform consists of the sensor unit, V2X unit, computing unit, and power unit, as shown in Figure 5.

During data collection, the camera resolution was 1080P (1920 × 1080) with a sampling rate of 30 Hz. Two radars were prepared for the experiment, including a high-resolution radar with 4 GHz bandwidth and a well-known 77 GHz Conti-ASR-408-21 radar. The sampling rate of the two radars was approximately 15 Hz. To verify the detection and localization accuracy of the proposed method, the detection result of high-resolution lidar was taken as the benchmark. Mechanical lidar can obtain different sampling frequencies by adjusting the rotation speed of the laser. To ensure data synchronization as synchronized as possible, the sampling frequency of the data collection system was set to 15 Hz.

The optimal sub-pattern assignment (OSPA) distance [57] is a comprehensive evaluation indicator that includes both localization and number errors, which can be taken as the evaluation criterion in this study.

(37) $$OSPA\left(d_{p}^{c}(X,Y)\right)={\left(\frac{1}{n}(\min_{\pi \in \Pi_{n}}\overset{m}{\underset{i=1}{\sum }}d^{c}{\left(x_{i},y_{\pi }(i)\right)}^{p}+c^{p}(n-m))\right)}^{1/p}$$

where the *X* and *Y* are two sets; *m* and *n* are the dimensions of two sets; *c* and *p* are the measure factor and distance order, respectively; *c* = 100, *p* = 2.

### 4.2. Tracking Algorithm Performance Analysis

#### 4.2.1. Tracking Experiment for Pedestrians

**Figure 6:**
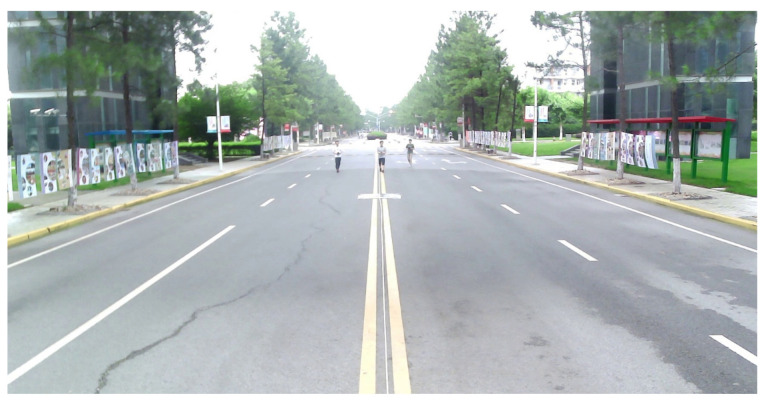
Pedestrian tracking experiment on a wide road.

**Figure 7:**
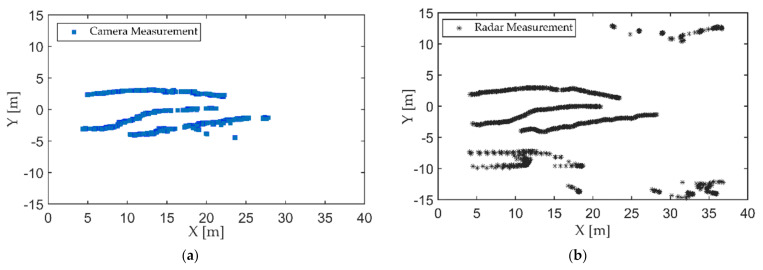
Detection results of targets: (**a**) camera; (**b**) radar.

**Figure 8:**
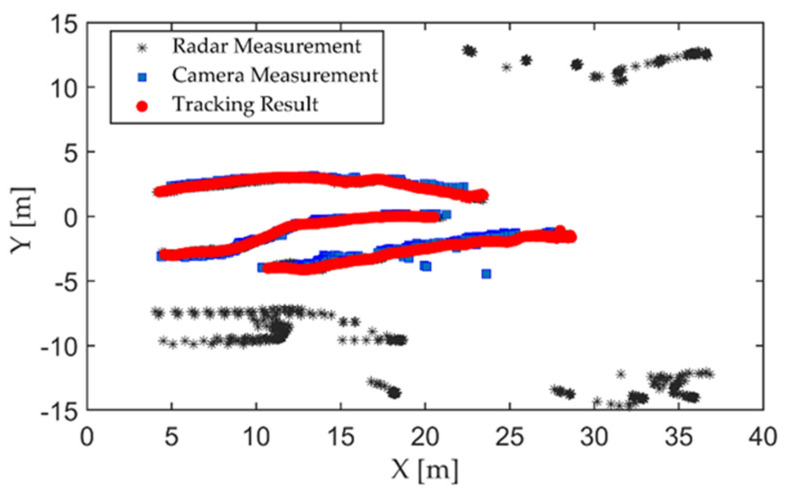
Tracking results of the proposed algorithm based on fusion data of the radar and camera.

The first experiment was carried out on a wide road on campus, as shown in Figure 6. The observed objects were three pedestrians walking along the predetermined trajectory, and there was no other traffic in the test area. Pedestrians are a vulnerable group of traffic participants, and accurate detection and localization are incredibly essential to ensure pedestrian safety. The high-resolution radar was used in the first experiment. Observations were collected from the intelligent roadside platform fixed on the middle of the road. The detection results of the camera and MMW radar were converted to a ground coordinate system with the observation platform as the origin, as shown in Figure 7. The tracking results based on radar and image data fusion are shown in Figure 8.

As shown in Figure 7 and Figure 8, both the camera and radar could detect pedestrians. Due to the range resolution and the localization error of the bounding box, there were some measurement errors and trajectory discontinuities in the detection results of the camera. The static targets regarded as interference measurements from the surrounding environment also appeared in radar detection results.

**Figure 9:**
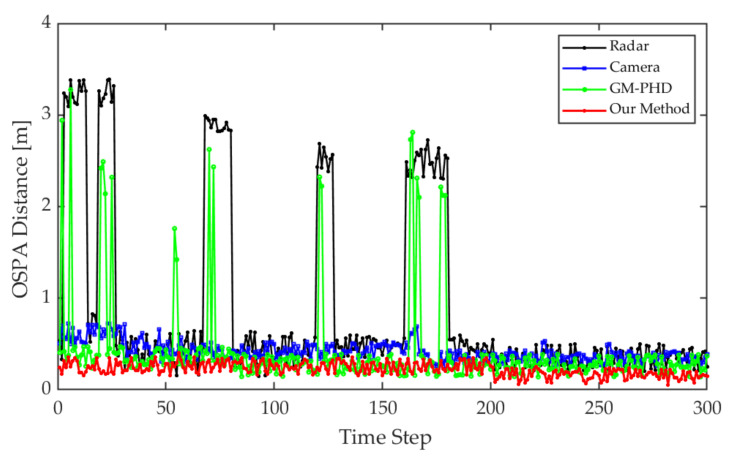
Optimal Sub-Pattern Assignment (OSPA) distance comparison for each tracking step.

The improved tracking algorithm could accurately extract the actual number of targets and ensure the continuity of the target trajectory based on fusion measurements of radar and image. To illustrate the improvements of the proposed method, we compared the single sensor’s tracking results and the initial GM-PHD method with our method. The single-sensor tracking algorithm adopted the tracking method in this study. All conditional assumptions of GM-PHD algorithm remained the same as [44]. The input of the GM-PHD algorithm was the fusion matrix obtained by Equation (12) without priori classification information and $P_{S,k}=0.98,\;P_{D,k}=0.95$. The detection results of the lidar with a higher localization accuracy were used as the benchmark. The OSPA distance is shown as Figure 9.

From the detection and tracking results of the camera, the proposed algorithm can reduce the localization errors caused by missed or false camera detections. That is because the elliptic threshold and attenuation function have the effects of filtering large errors and life-cycle maintenance in the tracking algorithm. However, this approach may have negative effects on radar tracking. Due to the lack of knowledge of target class information, the radar may incorrectly extract interference measurements as targets. By using the fusion data, the GM-PHD algorithm can use radar measurements for state updating when camera measurements were lost. However, the GM-PHD algorithm also does not consider the classification information, and other interference or similar measurements may be extracted as spooky targets in the pruning and merging step. The improved algorithm can maintain stable tracking based on the prior classification labels of the measurements and the optimal detection matching. Meanwhile, using the optimal measurement properties of the radar and camera for localization, the algorithm can significantly improve the localization accuracy. In the whole process, the average OSPA distance of the proposed algorithm is about 0.14 m, which is close to the localization accuracy of the lidar.

#### 4.2.2. Tracking Experiment for Cross Trajectory

**Figure 10:**
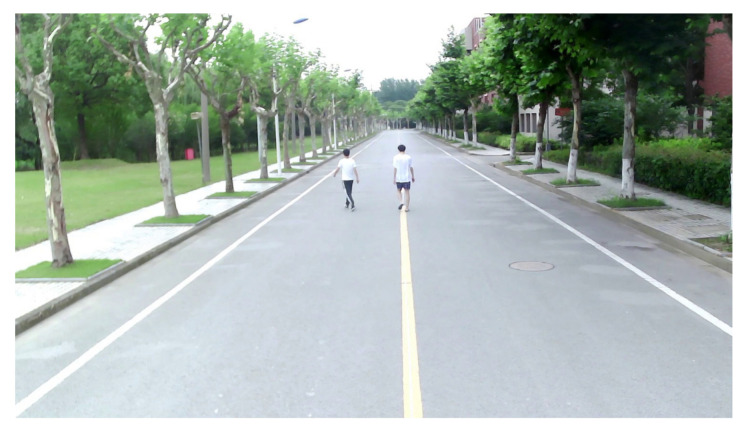
Pedestrian crosswalk tracking experiment.

The second test scenario was a trajectory-crossing tracking experiment for pedestrians, as shown in Figure 10. One of the pedestrians walked along the yellow line in a straight line, while the other pedestrians repeated the action of approaching and moving away, and the trajectories of the two pedestrians crossed several times. The crossing motion trajectories of pedestrians will cause occlusion, which is a tough problem in current target detection. All measurements were transformed to the ground coordinate system, and the algorithm performance comparison parameters remained the same as in the first experiment. A 77 GHz conti-ASR-408-21 radar, produced by Continental from Hanover, Germany, was used in this experiment. The detection and tracking results of targets and performance comparison are shown in the following.

**Figure 11:**
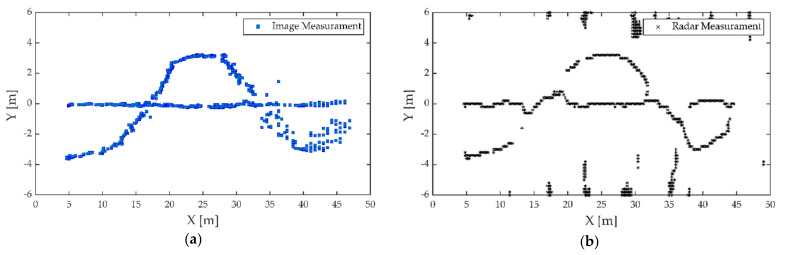
Detection results of targets: (**a**) camera; (**b**) radar.

**Figure 12:**
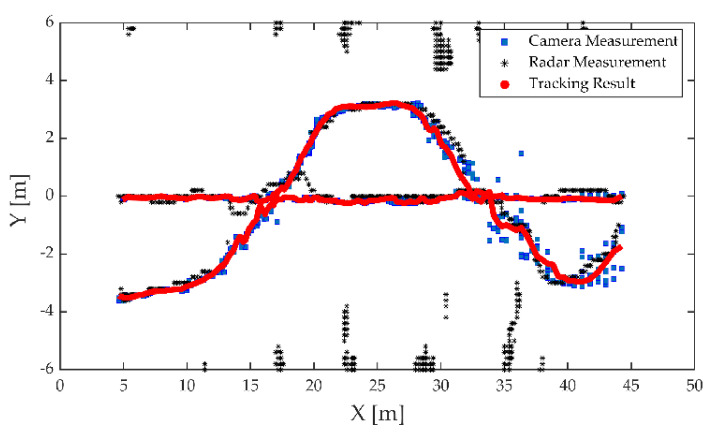
Tracking results for the proposed algorithm based on the radar and camera fusion data.

As can be seen in Figure 11, with an increase in distance, the detection accuracy of the image decreases significantly. On one hand, the swinging arm motion of the pedestrian caused a large variation in the scale of bounding box, resulting in localization errors increasing; on the other hand, the camera could not detect the occluded pedestrian, resulting in measurement loss. Meanwhile, the lateral distance resolution of this radar was about 0.2 m, and the radar could not accurately distinguish targets when pedestrians were moving closer due to the low azimuth resolution, resulting in a loss of measurement. Within 30 m, the radar measurements suffered serious loss, while the image measurements were more stable. However, the localization errors of image increased, while the radar performed better after 30 m. It is worth noting that radar and image measurements can complement each other better. Therefore, the proposed algorithm can continuously track the target trajectories based on the radar and camera fusion data, as shown in Figure 12.

From the tracking results for the camera, it can be seen that the proposed algorithm could ensure the tracking stability when the measurement was lost for a short time. As seen in the tracking results for the radar, the target measurements had been lost for too long, beyond the maximum life cycle that the algorithm could have maintained. Due to the lack of classification information in the radar measurements, the trajectory maintenance for spooky targets conversely increased the tracking error.

**Figure 13:**
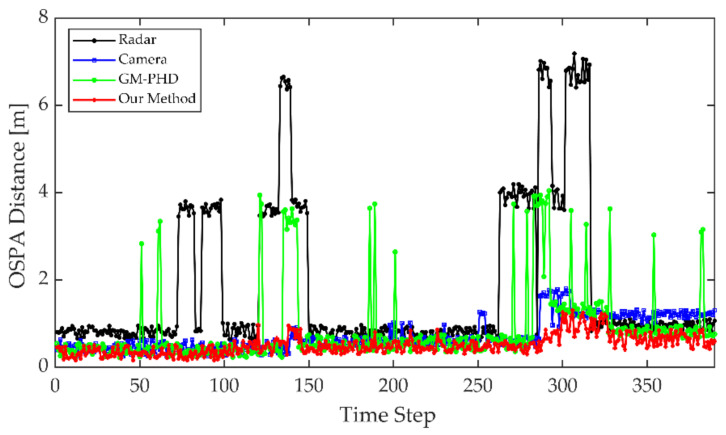
OSPA distance comparison for each tracking step.

Theoretically, when a sensor measurement is lost, the data fusion-based approach can use another sensor’s measurement for target tracking. However, the performance of the GM-PHD algorithm is heavily dependent on the observation quality. As shown is Figure 13, when the radar or camera measurements are lost or have large errors, the tracking error of the GM-PHD algorithm increases in the same way. Without the guidance of the target a priori category information, the GM-PHD algorithm could also extract ghost targets while only relying on the position information. The improved algorithm can use elliptic thresholds to exclude large errors or missing measurements that occur randomly and adjust the target update weights and survival periods adaptively by the missing tags and attenuation function. By smoothing the estimation error, the improved algorithm can maintain localization accuracy and tracking trajectory continuity. The prior classification information of the camera leads the algorithm to focus on valid targets to reduce the false alarm phenomenon. The experiment results finally demonstrated that the above approaches strengthened the robustness of the perception algorithm.

#### 4.2.3. Tracking Experiment for Vehicles

**Figure 14:**
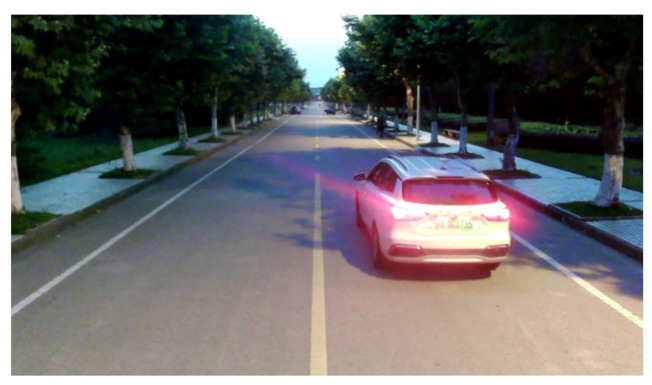
Vehicle tracking experiment in the evening.

**Figure 15:**
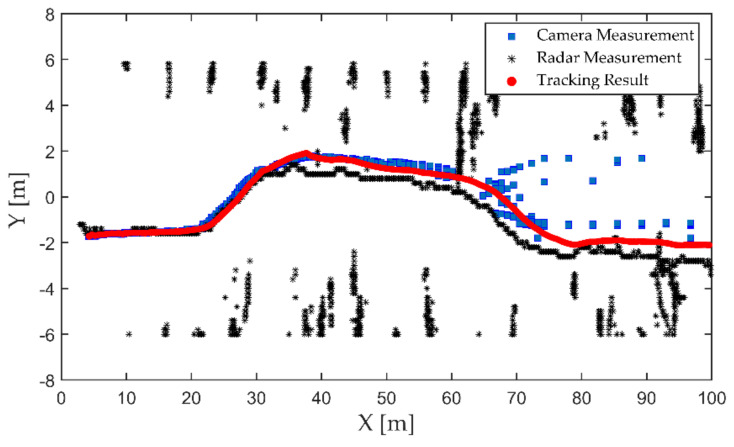
Vehicle detection and tracking results.

**Figure 16:**
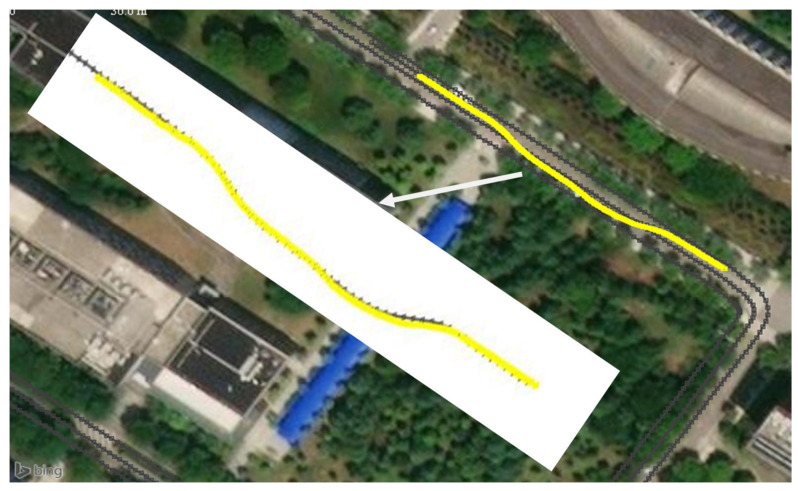
The tracking trajectory and global positioning system (GPS) trajectory in an high-definition (HD) map. The yellow line indicates the tracking result using the proposed algorithm, and the gray line is the measurements of the GPS system.

The third experiment was a vehicle-tracking experiment in the evening, as shown in Figure 14. The tested vehicle completed a lane change in a two-way lane. Due to the higher speed and stronger maneuverability of the vehicle, a farther monitoring distance was needed to give more reaction time to the intelligent networked cars or drivers. The tested vehicle was equipped with an inertial measurement unit (IMU) and a global positioning system (GPS) receiver to achieve centimeter-level localization through the real-time kinematic (RTK) technology. The GPS had a frequency of 10 Hz and a positioning accuracy of about 10 cm, the output of which was used as the benchmark for the tracking algorithm. The detection and tracking result of the vehicle is shown in Figure 15. A comparison of the tracking trajectory and GPS trajectory in a high-definition (HD) map is shown in Figure 16.

From the detection results of the camera and radar in Figure 15, it can be seen that the localization accuracy of both the camera and radar decreased significantly as the distance increased. The lack of longitudinal distance resolution of the camera is the main reason for its decreasing localization accuracy. Moreover, the radar’s low angular resolution caused a decrease in lateral localization accuracy. By using the optimal measurement attributes, our proposed algorithm could accomplish the target state update to improve the localization accuracy. Figure 16 shows that the tracking trajectory of the vehicle and GPS measurement trajectory almost coincided. The localization error was relatively small when the vehicle was driving in a straight line. The larger localization errors mainly occurred when the vehicle changed lanes, and the maximum lateral localization error was about 0.64 m. During the lane-changing process, the attitude of the vehicle relative to the sensor also changed continuously. The bounding-box size of the vehicle in the image detection algorithm and the reflected surface area of the echo signal in the radar detection underwent unpredictable nonlinear changes, which resulted in a bias in the lateral positioning of the vehicle. The proposed algorithm could achieve lane-level detection and localization of vehicles.

To sum up, three experiments demonstrated that the proposed algorithm had a significant improvement in localization accuracy and tracking stability. The proposed algorithm could realize the accurate localization of pedestrians within 50 m, and the lane-level localization of vehicles of at least 100 m.

### 4.3. System Validation

**Figure 17:**
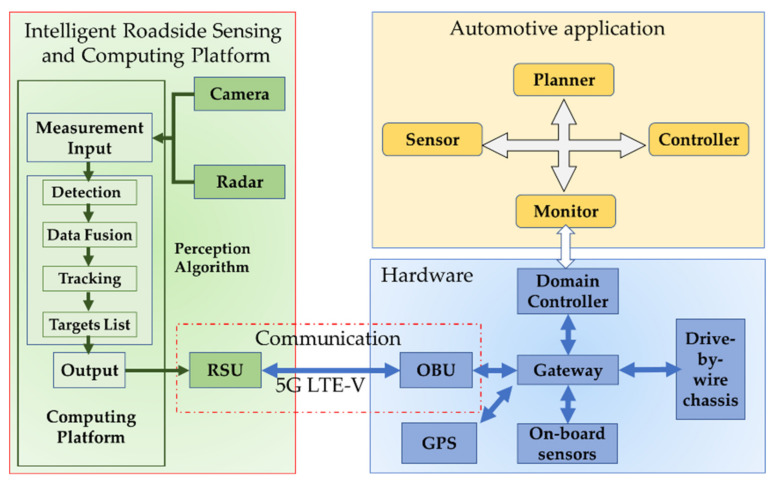
Main components of the system framework.

**Figure 18:**
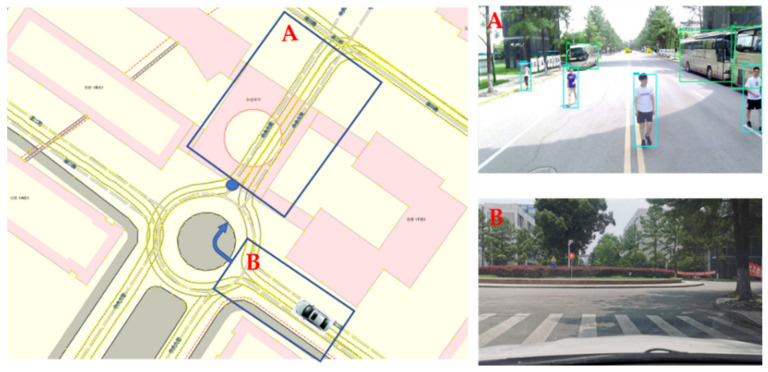
Test scenario for the vehicle-to-infrastructure (V2I) system. The left side is a HD map in vector format from OpenStreetMap. The blue marker is the placement of the roadside unit (RSU).

**Figure 19:**
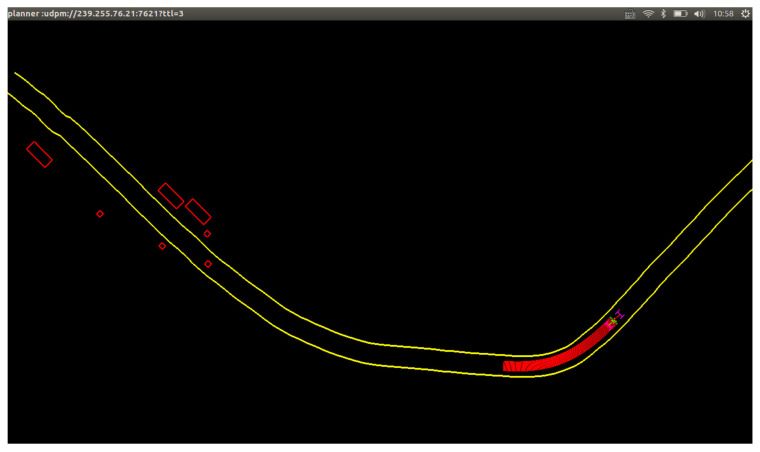
Visualization of the planner module. The yellow line is the driving route planned by the planner module. The red path indicates that the vehicle is slowing down or braking. Rectangles indicate vehicles. Smaller squares indicate pedestrians.

The system validation is a demonstration of a vehicle-to-infrastructure (V2I) application; the system framework is shown in Figure 17. The intelligent RSU used the camera and radar to complete the monitoring of the surrounding environment. The classification and location information of targets were packaged and broadcasted by the RSU to the OBU of the surrounding ICVs. Then the ICV generated a real-time road traffic situation map based on the received information and its HD map, which helped to complete the planning and decision-making in advance. The test scenario was a complex circular intersection on campus, as shown in Figure 18. Area A contained pedestrians and buses. The ICV was driving on the right turn road of area B, with tall trees on both sides. The data acquisition synchronization rate of the camera and the radar was 15 Hz, and the data transmission rate of RSU and the data reception rate of OUB were 10 Hz. For clear observation, we only visualized the planned path and target information, as shown in Figure 19.

The domain controller decoded the target information received by the OBU and loaded it into the planner. The high-precision location information and classification information of the target were displayed on the driving map display in real time. The planner of the ICV could plan the vehicle movement in real time based on the current traffic situation to ensure safe driving. In Figure 19, the ICV slowed down in advance to prevent a collision with pedestrians. Over-the-horizon perception allowed the ICV or drivers to anticipate the traffic situation in advance, and gave more time for decision-making. Therefore, this system can be used at intersections or in accident-prone areas, which is of great significance to reduce traffic collisions and improve traffic safety.

## 5. Conclusions

This study focused on roadside perception for ITSs. We proposed a multi-target detection and tracking algorithm based on the optimal property fusion of an MMW radar and camera. The framework could achieve classification, high accuracy localization, and trajectory tracking of targets in the observation field of view. The experiment results demonstrated that the proposed algorithm could improve the localization accuracy of targets and maintain the continuity of the trajectories. Meanwhile, this scheme realizes a high perception of confidence and stability with low-cost sensors, which is valuable for large-scale commercial applications to achieve traffic efficiency and safety. However, the whole perception process was actually a two-stage framework consisting of detection and tracking. All targets were regarded as points, ignoring the volume of targets, so that the algorithm could not track the pose-changing of targets with large volumes, such as trucks and buses. In our future research, we plan to design a one-stage end-to-end convolutional network to achieve high accuracy localization from the raw data of sensors. We will also use the 3-D bounding box to track the pose-changing and motion direction of targets.
